# Prognostic stratification in myocardial infarction using the modified CONUT score: a multidimensional biomarker from the MIMIC-IV cohort

**DOI:** 10.3389/fcvm.2025.1596575

**Published:** 2025-07-24

**Authors:** Weixu Yu, Yanting Luo, Qingyuan Cai, Jin Yang, Wanling Deng, Ruimin Dong, Xujing Xie

**Affiliations:** ^1^ Department of Cardiology, Zhongshan Hospital Xiamen University, Xiamen, China; ^2^ Department of Cardiology, Third Affiliated Hospital of Sun Yat-sen University, Guangzhou, China

**Keywords:** modified CONUT score, myocardial infarction prognosis, malnutrition assessment, MIMIC-IV database, mortality risk stratification

## Abstract

**Background:**

Malnutrition is increasingly recognized as a modifiable prognostic factor in myocardial infarction (MI), yet traditional nutritional assessment methods often fail to adequately capture lipid-related atherogenic risk. We introduced an innovative modified Controlling Nutritional Status (mCONUT) score, which replaces total cholesterol with non-HDL cholesterol, aiming to improve the stratification of atherosclerotic risk and to assess its prognostic utility in predicting MI outcomes.

**Methods:**

In this retrospective cohort study, we analyzed a total of 3,730 patients diagnosed with MI, extracted from the MIMIC-IV database, and stratified them into Normal, Mild, and Worse groups based on the mCONUT score. After performing 1:1:1 propensity score matching, we selected 993 patients for comparative analysis. Multivariable Cox proportional hazards models, adjusted for clinical and demographic confounders, were employed to evaluate all-cause mortality at 180 days and 1 year.

**Results:**

Among 993 MI patients, the median age was 75 years (IQR 68–82) with 58.4% males (*n* = 580). Malnutrition severity (mCONUT ≥6) stratified prognostic risk: Worse group demonstrated progressive mortality increases (180-day: 13.9% vs. Normal 7.85%, *p* = 0.037; 1-year: 31.4% vs. 21.2%, *p* = 0.011), alongside lower BMI, extended hospitalization, reduced hypertension, higher CKD incidence, and diminished revascularization (all *p* < 0.05). Multivariable analyses confirmed graded mortality risk: 180-day (Model 1: adjusted HR 1.58, *p* = 0.009; Model 2: HR 1.46, *p* = 0.031) and 1-year (Model 1: HR 1.61, *p* = 0.002; Model 2: HR 1.52, *p* = 0.008). Consistency across subgroups was observed, with heightened vulnerability in males, hypertensives (interaction *p* = 0.004), diabetes and non-white individuals.

**Conclusions:**

The mCONUT score has emerged as a robust multidimensional biomarker for predicting MI prognosis, with worse malnutrition (mCONUT ≥ 6) being significantly associated with a 46%–61% elevation in mortality risk, demonstrating a clear linear dose-response relationship. Routine screening and tailored nutritional interventions should be prioritized in modern MI management practices.

## 1 Introduction

Cardiovascular disease (CVD) continues to be the leading cause of death worldwide, accounting for an estimated 18 million fatalities each year, as reported by the World Health Organization (1). In China, CVD accounted for over 40% of all deaths in 2016 (2), a trend that has been further aggravated by population aging and the widespread adoption of unhealthy lifestyles. Myocardial infarction (MI), recognized as the most acute and life-threatening manifestation of CVD, continues to pose a substantial residual risk, even in the face of significant advances in reperfusion therapies and secondary prevention measures. Despite the decline in 1-year mortality from 30% in the pre-reperfusion era to 3%–8% in current clinical practice (3), the consistently high rate of recurrent cardiovascular events—especially among younger populations (4)—highlights an urgent need for innovative prognostic biomarkers. Growing evidence has identified malnutrition as a pivotal, modifiable risk factor significantly impacting cardiovascular outcomes.

Recent epidemiological studies have shown that 20%–50% of hospitalized patients and as many as 78% of critically ill individuals present with varying levels of malnutrition (5, 6). Among MI patients, almost half experience nutritional deficiencies, with 11.2% exhibiting moderate-to-severe malnutrition, as assessed using the Controlling Nutritional Status (CONUT) score (7). Originally proposed in 2005 (8), the CONUT score is a composite nutritional assessment tool incorporating serum albumin, total lymphocyte count, and total cholesterol (TC). Although validated across various cardiovascular populations (9–14), recent meta-analyses have highlighted significant limitations in its prognostic accuracy specifically for MI cohorts (15). Specifically, the inclusion of high-density lipoprotein cholesterol (HDL-C) within total cholesterol—a component known to be inversely associated with atherosclerotic risk (16)—may paradoxically reduce the score's ability to accurately capture atherogenic lipid burden.

To resolve this pathophysiological inconsistency, we established a modified CONUT (mCONUT) score by replacing TC with non-HDL cholesterol (TC minus HDL-C), aiming to enhance the score's specificity for identifying pro-atherogenic lipid profiles without compromising its capacity to assess nutritional status. This study seeks to explore the prognostic value of the mCONUT score in MI patients and to identify potential clinical applications for nutritional risk stratification within modern MI management strategies.

## 2 Materials and methods

### 2.1 MIMIC-IV database

This retrospective cohort study was conducted using the MIMIC-IV database (17), a comprehensive, publicly available dataset that provides detailed diagnostic, therapeutic, and nursing information for patients admitted to the emergency department and intensive care unit of Beth Israel Deaconess Medical Center (Boston, MA, USA).

The study protocol was reviewed and approved by the Institutional Review Boards of the Massachusetts Institute of Technology (Cambridge, MA, USA) and Beth Israel Deaconess Medical Center (Boston, MA, USA) (IRB approval number: 53015455). Given the study's retrospective design, the need for informed consent was waived by the Institutional Review Boards.

### 2.2 Study population

The study cohort was sourced from the MIMIC-IV database (v2.2), consisting of adult patients with a confirmed diagnosis of myocardial infarction (MI), identified using validated ICD-9 (410.00–410.92) and ICD-10 (I21-I22.9) codes. An initial screening process identified 9,698 MI cases, encompassing both ST-elevation (STEMI) and non-ST-elevation (NSTEMI) myocardial infarction subtypes. Systematic exclusion criteria were implemented as follows: (1) active malignancy (ICD codes C00-C97, *n* = 387), (2) decompensated liver cirrhosis (K70.3, K71.1, K72.1, K76.7, *n* = 77), (3) pregnancy (O09-O99, *n* = 2), and (4) incomplete nutritional or laboratory data (>30% missing, *n* = 5,502). Following exclusion, a final analytic cohort of 3,730 patients was established. Patients were stratified into three nutritional risk categories based on mCONUT thresholds: Normal (*n* = 2,122, 56.9%), Mild (*n* = 692, 18.6%), and Worse (*n* = 916, 24.5%). Baseline characteristics of the final cohort (3,730) are detailed in Supplementary Table.

**Figure 1:**
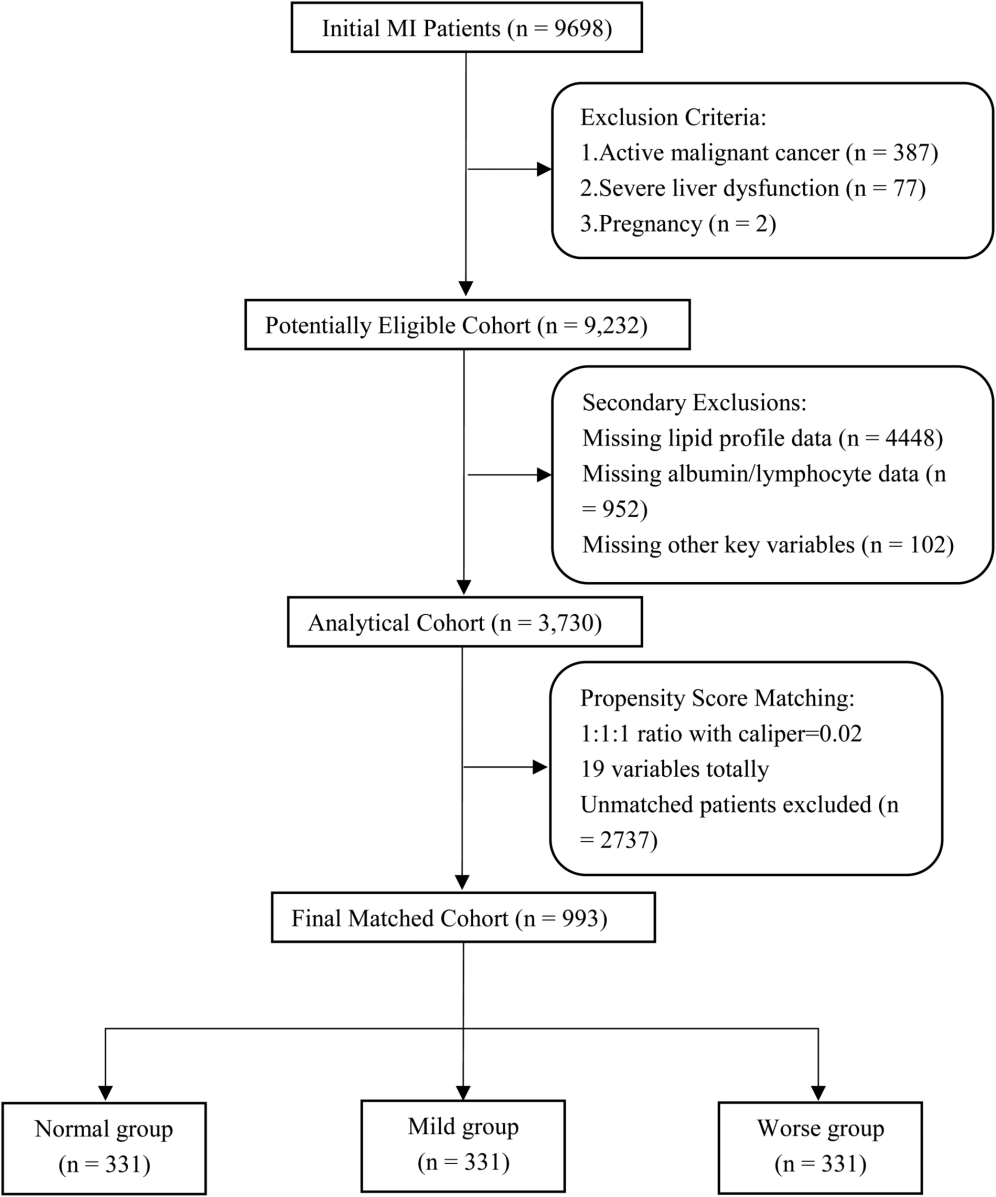
Search process and grouping of the included patients in the MIMIC-IV 2.2 database. MI, myocardial infarction;19 variables including age, gender, race, BMI (body mass index), comorbidities, procedures, medications and laboratory data.

To mitigate selection bias, 1:1:1 propensity score matching (PSM) with a caliper of 0.02 was applied, using age, gender, race, BMI, comorbidities, procedures, medications, and laboratory data as matching variables, resulting in well-balanced subgroups of 331 patients in each category (total *n* = 993). A detailed flowchart illustrating the patient selection process is presented in Figure 1.

### 2.3 Clinical data

For the final propensity-matched cohort, hospitalization records of MI patients were extracted using subject_id and hadm_id, which serve as unique identifiers for patient identity and hospital admission, respectively. For laboratory parameters measured repeatedly during hospitalization, only the initial test results recorded after admission were considered for analysis to ensure consistency.

Data extraction was systematically categorized into five domains: (1) Demographic Characteristics: age, sex, race, body mass index (BMI). (2) Comorbidities: hypertension, diabetes mellitus (DM), peripheral arterial disease (PAD), chronic kidney disease (CKD), stroke, atrial fibrillation (AF). (3) Therapeutic Interventions: percutaneous coronary intervention (PCI), coronary artery bypass grafting (CABG). (4) Medication Administration: vasoactive agents (VA), anti-platelet therapy (APT), beta-blockers (BB), renin-angiotensin system inhibitors (RASi), and statins. (5) Laboratory Parameters: complete blood count, comprehensive biochemical profile, and lipid panel.

Prognostic outcomes, including survival status and time-to-event data, were meticulously documented for subsequent analysis. Baseline characteristics of the study population are comprehensively summarized in Table 1.

**Table 1:** Baseline characteristics for final matched cohort divided by mCONUT.

Variables	Normal	Mild	Worse	*p*
	(*n* = 331)	(*n* = 331)	(*n* = 331)	
Demographic data				
Male, *n* (%)	192 (58.01)	209 (63.14)	179 (54.08)	0.06
Age, years	74.0 (67.0, 83.5)	74.0 (64.0, 84.0)	76.0 (65.0, 83.0)	0.705
BMI, kg/m^2^	27.80 (25.15, 31.95)	26.10 (24.10, 30.25)	26.10 (23.15, 29.80)	<.001
White, *n* (%)	75 (22.66)	87 (26.28)	97 (29.31)	0.149
LoH, days	7.0 (3.0, 14.0)	6.0 (3.0, 11.0)	9.0 (5.0, 14.0)	<.001
Comorbidities				
HT, *n* (%)	151 (45.62)	136 (41.09)	109 (32.93)	0.003
DM, *n* (%)	136 (41.09)	142 (42.90)	152 (45.92)	0.448
PAD, *n* (%)	63 (19.03)	57 (17.22)	69 (20.85)	0.494
CKD, *n* (%)	101 (30.51)	108 (32.63)	130 (39.27)	0.046
Stroke, *n* (%)	30 (9.06)	46 (13.90)	37 (11.18)	0.146
AF, *n* (%)	104 (31.42)	112 (33.84)	118 (35.65)	0.513
Procedures				
PCI, *n* (%)	163 (49.24)	181 (54.68)	145 (43.81)	0.02
CABG, *n* (%)	74 (22.36)	43 (12.99)	24 (7.25)	<.001
Medications				
VA, *n* (%)	118 (35.65)	98 (29.61)	109 (32.93)	0.252
APT, *n* (%)	304 (91.29)	314 (94.29)	309 (92.79)	0.377
BB, *n* (%)	285 (86.10)	301 (90.94)	293 (88.52)	0.149
RASi, *n* (%)	133 (40.18)	153 (46.22)	137 (41.39)	0.251
Statin, *n* (%)	297 (89.73)	297 (89.73)	292 (88.22)	0.77
Laboratory data				
Hb, g/dl	11.60 (10.25, 13.00)	11.20 (9.90, 12.40)	10.30 (8.85, 11.57)	<.001
Plt, 10^9^/L	224.0 (186.5, 280.0)	206.0 (156.0, 256.5)	214.0 (165.5, 277.0)	<.001
Scr, mg/dl	1.10 (0.90, 1.65)	1.20 (0.90, 1.85)	1.40 (1.00, 2.30)	<.001
TB, mg/dl	0.50 (0.30, 0.80)	0.50 (0.40, 0.80)	0.50 (0.30, 0.80)	0.805
Cl, mmol/L	102.0 (99.0, 105.0)	103.0 (100.0, 106.0)	103.0 (100.0, 106.0)	0.118
ALT, U/L	24.0 (16.0, 40.0)	25.0 (17.0, 43.0)	25.0 (15.0, 54.0)	0.698
Alb, g/dl	3.90 (3.70,4.20)	3.50 (3.30,3.70)	3.00 (2.70,3.20)	<.001
Lym, 10^9^/L	1.59 (1.27,2.05)	1.24 (0.92,1.68)	1.08 (0.85,1.55)	<.001
HDL, mg/dl	48.00 (36.00,56.50)	44.00 (36.00,55.50)	44.00 (34.00,54.00)	0.021
TC, mg/dl	170.0 (143.0,202.5)	146.0 (122.0,176.0)	133.0 (110.0,169.0)	<.001
NewTC, mg/dl	48.00 (36.00,56.50)	44.00 (36.00,55.50)	44.00 (34.00,54.00)	<.001
mCONUT	2.0 (1.0,3.0)	4.00 (4.0,5.0)	7.0 (6.0,8.0)	<.001
Prognosis				
180d-d, *n* (%)	26 (7.85)	33 (9.97)	46 (13.90)	0.037
365d-d, *n* (%)	70 (21.15)	88 (26.59)	104 (31.42)	0.011

BMI, body mass index; LoH, length of hospital stay; HT, hypertension; DM, diabetes mellitus; PAD, peripheral artery diseases; CKD, chronic kidney disease; AF, atrial fibrillation; PCI, percutaneous coronary intervention; CABG, coronary artery bypass grafting; VA, vasoactive agents; APT, antiplatelet therapy; BB, beta blockers; RASi, renin-angiotensin-aldosterone system inhibitors; Hb, hemoglobin; Plt, platelet; Scr, serum creatinine; TB, total bilirubin; Cl, chlorine; ALT, alanine aminotransferase; Alb, albumin; Lym, lymphocyte count; HDL, high-density lipoprotein cholesterol; TC, total cholesterol; NewTC, non-high-density lipoprotein cholesterol (calculated as TC—HDL-C); mCONUT, modified controlling nutritional status scoring; 180d-d, death of 180 days after admission; 365d-d, death of 1 year after admission.

### 2.4 Modified CONUT score and follow-up endpoints

The mCONUT score is an optimized nutritional assessment tool that replaces total cholesterol (TC) with non-HDL cholesterol (TC minus HDL-C), addressing a critical pathophysiological inconsistency in MI cohorts. By eliminating HDL-C—a protective, anti-atherogenic lipid—the mCONUT score focuses exclusively on pro-atherogenic lipids (e.g., LDL-C, remnant cholesterol), thereby enhancing its capacity to evaluate malnutrition in the context of atherosclerosis-driven prognosis.

Optimal thresholds for albumin, lymphocyte count, and non-HDL cholesterol were determined using restricted cubic spline (RCS) analysis (Supplementary Figures), ensuring data-driven cutoff values. Each of the three biomarkers demonstrated statistically significant nonlinear associations with mortality (*p* < 0.001 for knot transitions), with inflection points objectively determined as optimal cutoffs.

The mCONUTscore is calculated by summing points assigned to three laboratory parameters: albumin (>3.7 g/dl = 0; 3.4–3.7 = 2; 3.0–3.4 = 4; <3.0 = 6), lymphocyte count (>1,500/μl = 0; 1,100–1,500 = 1; 700–1,100 = 2; <700 = 3), and non-HDL cholesterol (>110 mg/dl = 0; 90–110 = 1; 60–90 = 2; <60 = 3). The total score classifies nutritional risk: 0–3 indicates mild malnutrition, 4–5 moderate malnutrition, and 6–12 severe malnutrition, as outlined in Table 2. The primary endpoints were defined as 180-day and 1-year all-cause mortality following hospital admission.

**Table 2:** Modified controlling nutritional Status scoring (mCONUT).

Indices	Nutritional Risk			
	Normal	Mild	Worse	
Alb, g/dl	>3.7 (0)	3.4–3.7 (2)	3.0–3.4 (4)	<3.0 (6)
Lym, u/μl	>1.5 (0)	1.1–1.5 (1)	0.7–1.1 (2)	<0.7 (3)
Non-HDL TC, mg/dl	>110 (0)	90–110 (1)	60–90 (2)	<60 (3)
Total	0–3	4–5	6–12	

Alb, albumin; Lym, lymphocyte count; Non-HDL TC, non-high-density lipoprotein total cholesterol.

### 2.5 Statistical analysis

The distribution of continuous variables was evaluated using the Kolmogorov–Smirnov test, with a *p*-value threshold of <0.1 indicating deviation from normality. Normally distributed data were reported as mean ± standard deviation (SD), while non-normally distributed data were presented as median with interquartile range [IQR]. Categorical variables were described as frequencies and percentages.

Group comparisons were conducted using analysis of variance (ANOVA) for normally distributed continuous variables, Kruskal–Wallis tests for non-normally distributed continuous variables, and chi-square (*χ*²) tests for categorical variables, with a two-tailed significance level of *α* = 0.05. Multivariable Cox proportional hazards regression models employing backward stepwise selection (retention criterion *p* < 0.05) were utilized to assess the association between malnutrition categories and clinical outcomes. A hierarchical adjustment approach was employed: Model 1 accounted for demographic characteristics (age, sex, race, and BMI), while Model 2 further adjusted for key clinical covariates, including hospitalization duration, hypertension, diabetes, PCI, VA, BB, hemoglobin, and serum creatinine levels.

Temporal survival distributions were visualized using Kaplan–Meier survival curves, and differences between stratified groups were statistically assessed using the log-rank test. To ensure the robustness of findings, prespecified subgroup analyses were conducted across clinically relevant strata, including age (≥75 vs. <75 years), sex, race, hypertension, diabetes, CKD, history of stroke, and revascularization status. Sensitivity analyses were performed using univariate Cox regression models, with interaction terms assessed to identify potential effect modifiers. All statistical analyses were performed using R software (version 4.4.2).

## 3 Results

### 3.1 Baseline characteristics

Table 1 presents baseline characteristics of the 993 MI patients stratified by mCONUT nutritional status. The Worse group (*n* = 331) demonstrated significantly lower BMI (26.1 vs. 27.8/26.1 kg/m^2^, *p* < 0.001), prolonged hospitalization (9.0 vs. 7.0/6.0 days, *p* < 0.001), and paradoxically reduced hypertension prevalence (32.9% vs. 45.6%/41.1%, *p* = 0.003) compared to Normal/Mild groups. This cohort exhibited higher CKD rates (39.3% vs. 30.5%/32.6%, *p* = 0.046), lower revascularization (PCI: 43.8% vs. 49.2%/54.7%; CABG: 7.3% vs. 22.4%/13.0%, both *p* < 0.05), and worse hematological profiles (hemoglobin, platelets, creatinine, albumin, lymphocytes; all *p* < 0.001). Nutritional status did not influence age, sex, ethnicity, or medication use. Mortality progressively increased with malnutrition severity (180-day: 13.9% Worse vs. 7.85% Normal, *p* = 0.037; 1-year: 31.4% vs. 21.2%, *p* = 0.011).

### 3.2 Malnutrition and prognosis

During the 180-day follow-up period, 105 fatalities (10.57%) occurred, demonstrating a graded mortality increase across nutritional strata: Normal group (*n* = 26, 7.85%), Mild group (*n* = 33, 9.97%), and Worse group (*n* = 46, 13.90%). Univariate Cox regression (Table 3) demonstrated significantly elevated mortality risk in the Worse vs. Normal group (HR 1.59, 95% CI 1.13–2.23; *p* = 0.008), whereas the Mild group exhibited no significant difference (HR 1.15, 95% CI 0.80–1.66; *p* = 0.435). Following multivariable adjustment for confounders, the Worse group maintained significantly heightened mortality risk. Model 1 (adjusted for age, sex, race, BMI) yielded an HR of 1.58 (95% CI 1.12–2.23; *p* = 0.009). Model 2 (further adjusted for hospitalization duration, hypertension, diabetes, PCI, VA, BB, hemoglobin, and creatinine) revealed a hazard ratio of 1.46 (95% CI 1.10–2.19; *p* = 0.031).

**Table 3:** Cox proportional hazards regression analysis of mortality risk by mCONUT score.

Prognosis	Group	Unadjusted	*p*	Model 1^a^	*p*	Model 2^b^	*p*
		HR (95% CI)		HR (95% CI)		HR (95% CI)	
180-day mortality	Normal	Reference		Reference		Reference
	Mild	1.15 (0.80–1.66)	0.435	1.17 (0.81–1.68)	0.41	1.21 (0.84–1.74)	0.310
	Worse	1.59 (1.13–2.23)	0.008	1.58 (1.12–2.23)	0.009	1.46 (1.10–2.19)	0.031
1-year mortality	Normal	Reference		Reference		Reference	
	Mild	1.28 (0.93–1.75)	0.124	1.30 (0.95–1.78)	0.104	1.34 (0.93–1.83)	0.07
	Worse	1.59 (1.17–2.15)	0.003	1.61 (1.19–2.18)	0.002	1.52 (1.12–2.07)	0.008

^a^ Model 1: Adjusted for age, sex, race, and BMI.

^b^ Model 2: Additionally adjusted for hospitalization duration, hypertension, diabetes, PCI, vasoactive agents, β-blockers, hemoglobin, and creatinine.

**Figure 2:**
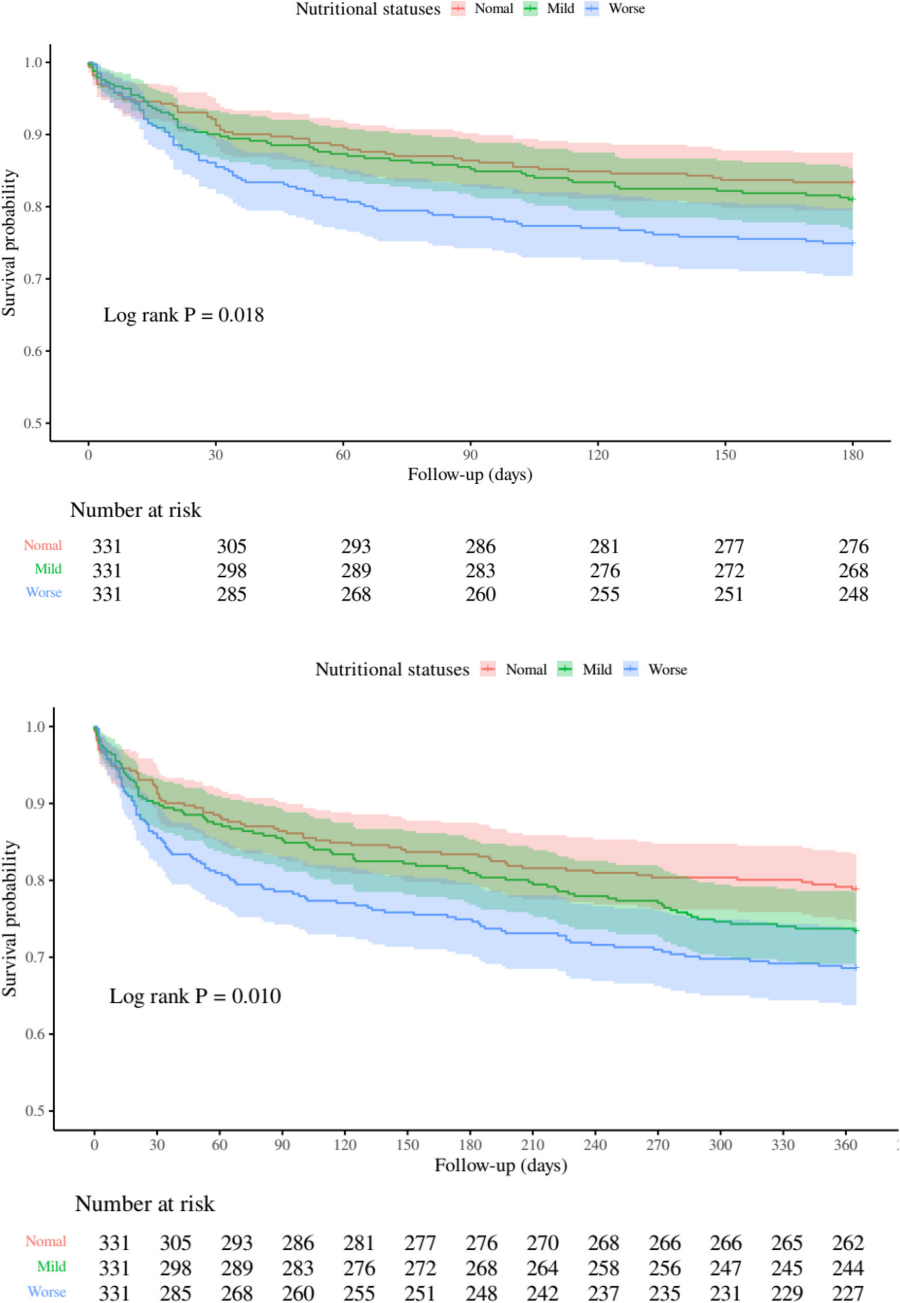
Kaplan-Meier curves depicting all-cause mortality risk at 180 days (top) and 1 year (bottom) across nutritional status categories.

At the 1-year follow-up, 262 deaths (26.38%) were documented: Normal group (*n* = 70, 21.15%), Mild group (*n* = 88, 26.59%), and Worse group (*n* = 104, 31.42%). Univariate analysis indicated a 59% elevated mortality risk in the Worse group (95% CI 1.17–2.15, *p* = 0.003), with Model 1 showing a 61% increase (95% CI 1.19–2.18; *p* = 0.002) and Model 2 a 52% increase (95% CI 1.12–2.07; *p* = 0.008). Kaplan–Meier analysis (Figure 2) corroborated these observations, evidencing significant survival curve divergence at 180 days (log-rank *p* = 0.018) and 1 year (log-rank *p* = 0.010).

**Figure 3:**
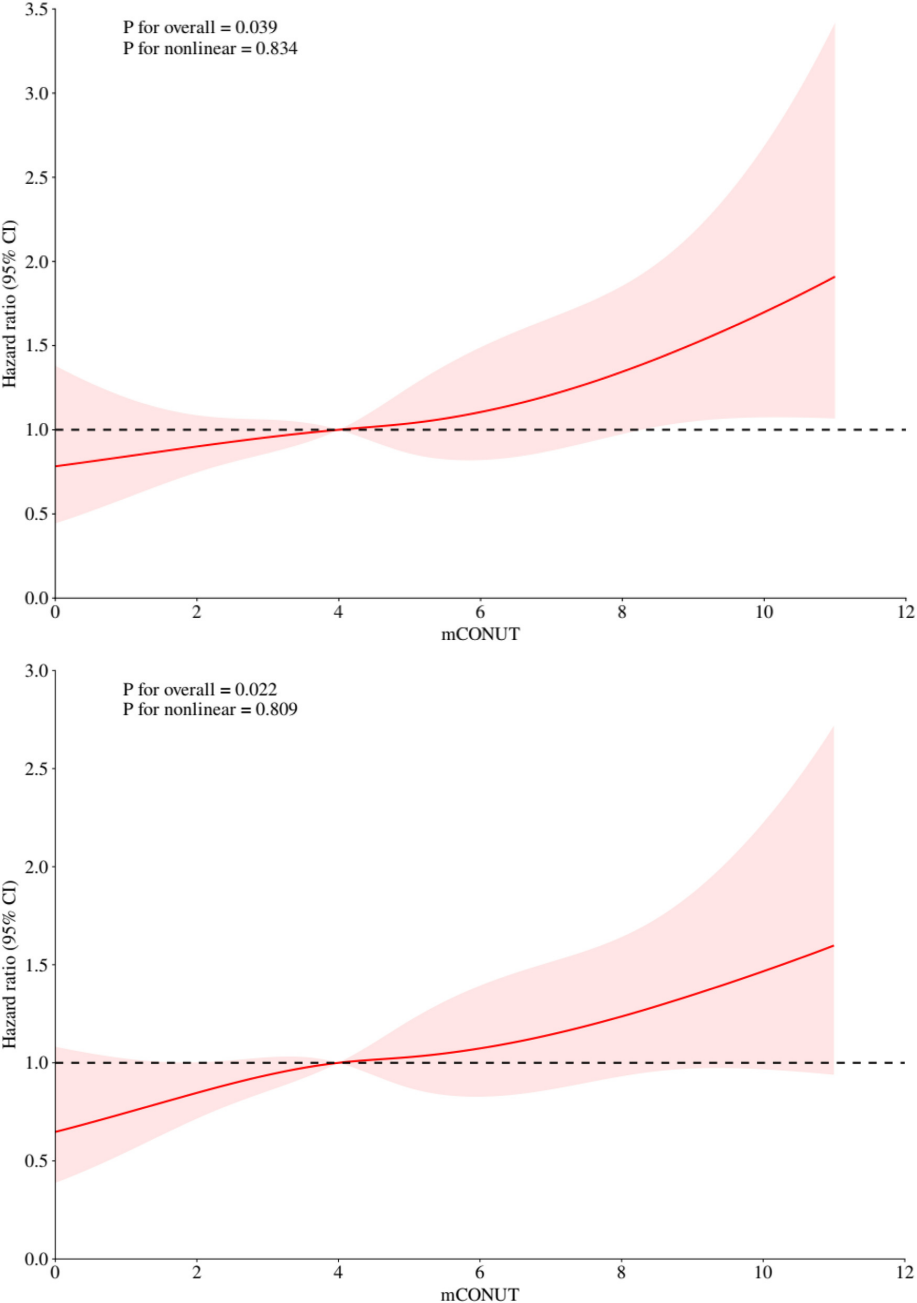
Restricted cubic spline analyses illustrating the association between mCONUT scores and all-cause mortality risk at 180 days (top) and 1 year (bottom).

The restricted cubic spline analysis (Figure 3) demonstrated significant linear associations between mCONUT scores and mortality risk at 180 days (p-overall = 0.039) and 360 days (p-overall = 0.022), with no evidence of nonlinearity (p-nonlinear = 0.834 and 0.809, respectively). Hazard ratio curves exhibited consistent dose-response patterns devoid of inflection points or threshold effects, supporting a progressive mortality risk elevation with increasing mCONUT scores during both follow-up intervals.

### 3.3 Subgroup analyses

**Figure 4:**
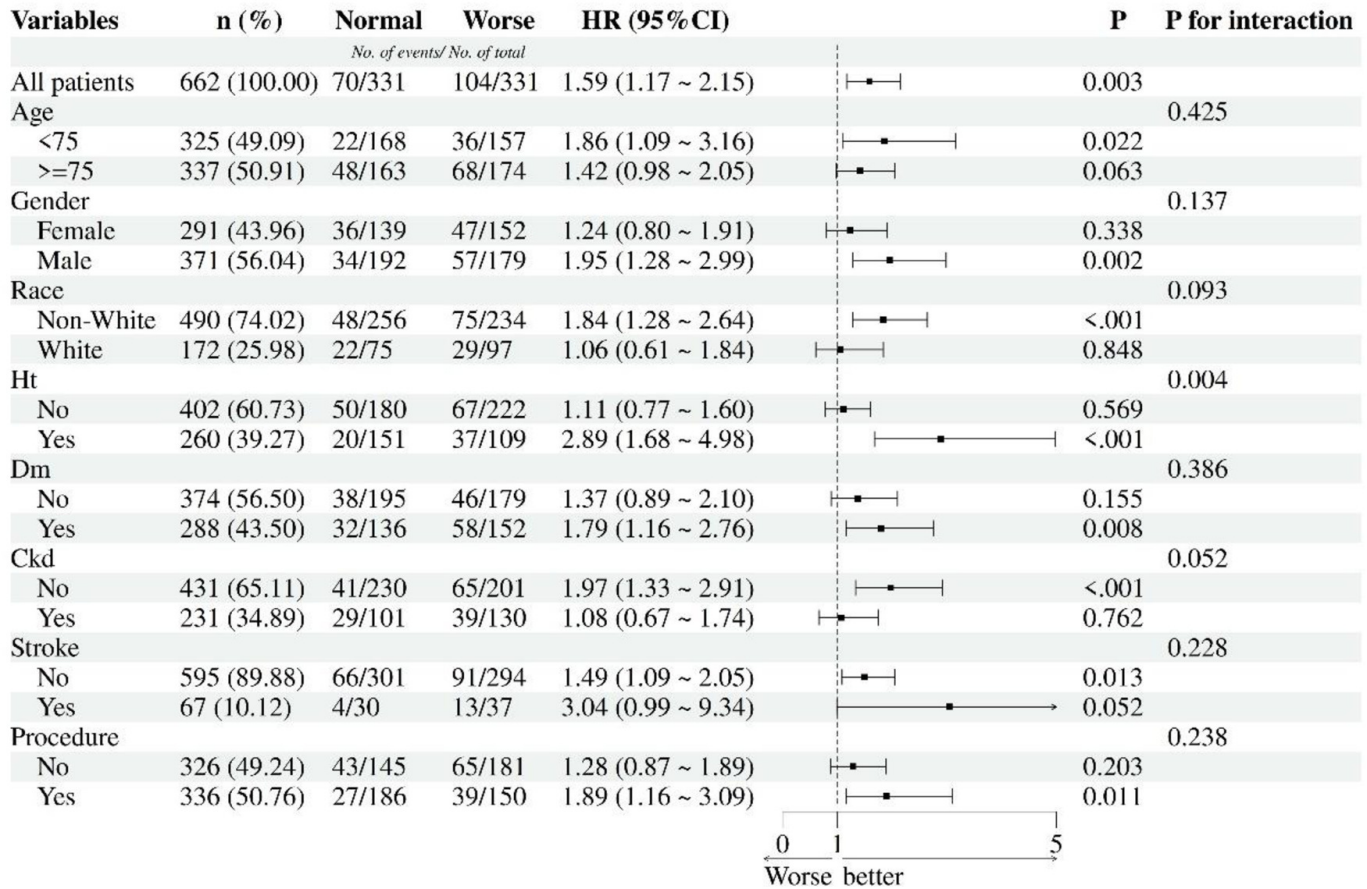
Subgroup analyses for nutritional statuses and 1-year all-cause mortality. HT, hypertension; DM, diabetes mellitus; CKD, chronic kidney diseases; Procedures including PCI, percutaneous coronary intervention; CABG, coronary artery bypass grafting.

Subgroup analyses (Figure 4) using univariate cox regression revealed differential mortality risks associated with worse malnutrition (mCONUT-defined) across clinical and demographic strata. Patients <75 years exhibited an 86% increased mortality risk (HR 1.86, 95% CI 1.09–3.16; *p* = 0.022), contrasting with non-significant findings in those ≥75 years (HR 1.42, 95% CI 0.98–2.05; *p* = 0.063). Males demonstrated markedly elevated risk (HR 1.95, 95% CI 1.28–2.99; *p* = 0.002), while females showed no significant association (HR 1.24, 95% CI 0.80–1.91; *p* = 0.338). Non-white individuals experienced an 84% higher mortality (HR 1.84, 95% CI 1.28–2.64; *p* < 0.001), unlike white counterparts (HR 1.06, 95% CI 0.61–1.84; *p* = 0.848).

Hypertensive patients displayed a striking 2.89-fold risk elevation (95% CI 1.68–4.98; *p* < 0.001), whereas non-hypertensives showed no association (HR 1.11, 95% CI 0.77–1.60; *p* = 0.569). Diabetics exhibited increased mortality (HR 1.79, 95% CI 1.16–2.76; *p* = 0.008), contrasting with non-diabetics (HR 1.37, 95% CI 0.89–2.10; *p* = 0.155). Notably, CKD-free patients demonstrated significant risk (HR 1.97, 95% CI 1.33–2.91; *p* < 0.001), unlike CKD patients (HR 1.08, 95% CI 0.67–1.74; *p* = 0.762). Stroke history modified risk association: Non-stroke patients showed significant risk elevation (HR 1.49, 95% CI 1.09–2.05; *p* = 0.013), while stroke survivors demonstrated marginally significant increased mortality (HR 3.04, 95% CI 0.99–9.34; *p* = 0.052). Revascularized patients (PCI/CABG) showed elevated risk (HR 1.89, 95% CI 1.16–3.09; *p* = 0.011), while non-revascularized individuals trended toward significance (HR 1.28, 95% CI 0.87–1.89; *p* = 0.203). Significant interaction effects emerged for hypertension (*p* for interaction = 0.004), with other subgroups showing non-significant interactions.

## 4 Discussion

Our study presents three key findings from the comprehensive analysis of the MIMIC-IV cohort: (1) Malnutrition affects 43.1% of MI patients (mCONUT ≥ 4), with 24.5% exhibiting severe malnutrition (mCONUT ≥ 6); (2) The mCONUT score is a robust prognostic tool, with severe malnutrition independently predicting a 46%–58% higher risk of mortality at 180 days and a 52%–61% increased risk at 1 year after multivariable adjustment; (3) Nonlinear analysis demonstrates a dose-response relationship between mCONUT scores and mortality risk, validated through restricted cubic spline curves. These findings not only reinforce but also extend previous research on nutritional indices, marking three significant advancements in the field.

Nutritional assessments in clinical practice are often subjective, but objective tools such as the Subjective Global Assessment (SGA) (18) and Mini Nutritional Assessment (MNA) (19). Other indices like the Prognostic Nutritional Index (PNI) (20), based on lymphocyte count and serum albumin levels, and the Geriatric Nutritional Risk Index (GNRI) (21), which uses albumin and body weight, also serve as reliable indicators of malnutrition. A PNI below 45 or a GNRI under 98 has been associated with adverse outcomes, including increased mortality. Studies have linked lower serum albumin levels and malnutrition to worse outcomes in coronary heart disease, including higher risks of acute heart failure and cardiogenic shock in ACS patients (22, 23). The “Cholesterol Paradox” further suggests that low LDL cholesterol, potentially associated with malnutrition, correlates with poorer long-term outcomes (25–28). Malnutrition can induce immune dysfunction, as energy depletion impairs immune system activation, which is energy-intensive. Lymphocyte count, reflecting both immune function and nutritional status, has been identified as an independent risk factor for poor prognosis in coronary artery disease, particularly in ACS (30, 31). The CONUT score, which evaluates albumin, total cholesterol (TC), and lymphocyte count, is widely used to assess nutritional status in cardiovascular diseases (32–38). Our study introduces a modified version, the mCONUT score, which replaces total cholesterol with non-HDL cholesterol for improved predictive accuracy in MI patients, particularly those with severe malnutrition.

In our cohort, patients with mCONUT scores ≥6 exhibited longer hospital stays and worse in-hospital outcomes, consistent with previous studies (7, 37, 38) linking severe malnutrition to hemodynamic instability and an exaggerated inflammatory response (39). These patients also faced significantly higher risks of all-cause mortality. Notably, the mortality risk gradient was most pronounced in male patients aged <75 years undergoing procedures and those with multiple comorbidities, particularly hypertension, diabetes, and stroke. This aligns with other research showing that ACS patients with moderate to severe malnutrition face a 2.02- to 3.65-fold increase in mortality risk (7, 40, 41).

The association between the mCONUT score and prognosis likely operates through two interconnected pathways: metabolic depletion and immunoinflammatory dysregulation. By incorporating non-HDL cholesterol, a superior marker for atherosclerotic risk (42), instead of total cholesterol, the mCONUT score better reflects lipid metabolism abnormalities in malnourished MI patients. Hypoalbuminemia (albumin <3.5 g/dl) in severe malnutrition impairs antioxidant defenses and nitric oxide bioavailability, exacerbating myocardial ischemia (43). Concurrently, lymphocytopenia (lymphocyte count <1.5 × 10³/μl) reflects immune system collapse, allowing uncontrolled inflammation after an infarction (44). This results in a pathogenic cycle of energy deficit, immune exhaustion, and persistent inflammation, as evidenced by elevated neutrophil-to-lymphocyte ratio (NLR) and C-reactive protein (CRP) levels in MI patients (45). Recent metabolomic studies (46) have further identified malnutrition-induced depletion of branched-chain amino acids, critical for cardiac energy production, as a potential driver of ventricular remodeling.

Addressing malnutrition with clinical interventions can improve patient outcomes. Nutritional support, such as oral supplements, has been shown to enhance muscle strength and recovery in malnourished patients with cardiopulmonary diseases like ACS. Moreover, nutritional interventions can reduce mortality and improve post-discharge recovery in these patients (47, 48). Our study confirms that the modified CONUT score is a multifaceted prognostic biomarker, integrating nutritional, metabolic, and immunological risks for MI patients. For clinical implementation, we recommend: (1) systematic mCONUT screening at admission to stratify high-risk patients; (2) dynamic monitoring of albumin (>3.8 g/dl) and lymphocyte count (>1.8 × 10³/μl); and (3) post-discharge nutritional protocols to mitigate the inflammation-malnutrition cycle. These recommendations underscore the potential of mCONUT as a modifiable determinant of adverse outcomes in MI management.

Despite rigorous adjustments, our study may still be subject to residual confounding due to unmeasured inflammatory markers (e.g., IL-6) and socioeconomic factors. Additionally, the single-center design limits generalizability, though the demographic diversity of the MIMIC-IV 2.2 cohort partially mitigates this concern. While we have established temporal associations, further validation through intervention studies targeting malnutrition is needed to establish causality.

## Data Availability

The raw data supporting the conclusions of this article will be made available by the authors, without undue reservation.

## Supplementary material

Supplementary Figures Content: Restricted Cubic Spline (RCS) analyses for albumin, lymphocyte count, and non-HDL cholesterol cutoff determination.
