# Supplementary material for: Prognostic stratification in myocardial infarction using the modified CONUT score: a multidimensional biomarker from the MIMIC-IV cohort
Source: Front Cardiovasc Med. 2025 Jul 24;12:1596575. doi: 10.3389/fcvm.2025.1596575 (PMC12328424; doi:10.3389/fcvm.2025.1596575)
Supplement: Supplementary Figures — Content: Restricted Cubic Spline (RCS) analyses for albumin, lymphocyte count, and non-HDL cholesterol cutoff determination. [file Table1.docx]

| Supplementary Table. Baseline characteristics of the cohort before propensity score matching. | | | | | |
| --- | --- | --- | --- | --- | --- |
| Variables | Normal (n = 2122) | Mild (n = 692) | Worse (n = 916) | *P* | |
|  |  |  |  |  |  |
| **Demographic data** | | | | | |
| Male, n (%) | 1297 (61.12) | 419 (60.55) | 509 (55.57) | **0.014** | |
| Age, years | 69.0 (59.0,80.0) | 73.0 (64.0,83.0) | 74.0 (64.0,83.0) | **<.001** | |
| BMI, kg/m^2^ | 27.80 (25.60,32.10) | 26.40 (24.30,31.10) | 26.10 (23.58,30.60) | **<.001** | |
| White, n (%) | 335 (15.79) | 167 (24.13) | 304 (33.19) | **<.001** | |
| LoH, days | 4.00 (2.00,8.00) | 6.00 (3.00,11.00) | 9.00 (5.00,15.00) | **<.001** | |
| **Comorbidities** | | | | | |
| HT, n (%) | 1032 (48.63) | 276 (39.88) | 305 (33.30) | **<.001** | |
| DM, n (%) | 816 (38.45) | 288 (41.62) | 390 (42.58) | 0.067 | |
| PAD, n (%) | 261 (12.30) | 109 (15.75) | 190 (20.74) | **<.001** | |
| CKD, n (%) | 527 (24.84) | 223 (32.23) | 350 (38.21) | **<.001** | |
| Stroke, n (%) | 191 (9.00) | 89 (12.86) | 113 (12.34) | **0.002** | |
| AF, n (%) | 491 (23.14) | 238 (34.39) | 325 (35.48) | **<.001** | |
| **Procedures** | | | | | |
| PCI, n (%) | 1384 (65.22) | 383 (55.35) | 393 (42.90) | **<.001** | |
| CABG, n (%) | 300 (14.14) | 88 (12.72) | 79 (8.62) | **<.001** | |
| **Medications** | | | | | |
| VA, n (%) | 404 (19.04) | 194 (28.03) | 360 (39.30) | **<.001** | |
| APT, n (%) | 1926 (90.76) | 635 (91.76) | 805 (87.88) | **0.001** | |
| BB, n (%) | 1926 (90.76) | 635 (91.76) | 805 (87.88) | **0.016** | |
| RASi, n (%) | 1367 (64.42) | 424 (61.27) | 432 (47.16) | **<.001** | |
| Statin, n (%) | 977 (93.17) | 619 (89.45) | 811 (88.54) | **<.001** | |
| **Laboratory data** | | | | | |
| Hb, g/dL | 12.30 (10.90,13.70) | 11.10 (9.70,12.60) | 10.30 (8.90,11.70) | **<.001** | |
| Plt, 10^9/L | 218.00 (178.00,263.00) | 200.00 (155.00,251.25) | 208.00 (158.00,275.00) | **<.001** | |
| Scr, mg/dL | 1.00 (0.80,1.40) | 1.20 (0.90,1.90) | 1.40 (0.90,2.30) | **<.001** | |
| TB, mg/dL | 0.61 ± 0.68 | 0.72 ± 1.18 | 0.74 ± 1.37 | **<.001** | |
| Cl, mmol/L | 102.39 ± 4.72 | 102.20 ± 5.32 | 102.69 ± 6.56 | 0.170 | |
| ALT, U/L | 44.03 ± 87.72 | 63.37 ± 169.50 | 94.99 ± 298.64 | **<.001** | |
| Alb, g/dl | 4.00 (3.80,4.30) | 3.50 (3.30,3.70) | 2.95 (2.70,3.20) | **<.001** | |
| Lym, 10^9/L | 1.63 (1.28,2.10) | 1.29 (0.93,1.68) | 1.08 (0.83,1.51) | **<.001** | |
| HDL, mg/dL | 46.00 (38.00,56.00) | 45.00 (36.00,56.00) | 45.00 (35.00,55.00) | **<.001** | |
| TC, mg/dL | 176.00 (149.00,206.00) | 143.50 (119.75,174.00) | 135.50 (111.00,170.00) | **<.001** | |
| NewTC, mg/dL | 125.00 (102.00,157.00) | 94.00 (76.00,124.25) | 89.00 (68.00,119.00) | **<.001** | |
| mCONUT | 1.0 (0.0,2.0) | 4.00 (4.0,5.0) | 7.00 (6.0,8.0) | **<.001** | |
| **Prognosis** | | | | | |
| Death, n (%) | 284 (13.38) | 167 (24.13) | 321 (35.04) | **<.001** | |
| BMI, body mass index; LoH, length of hospital stay; HT, hypertension; DM, diabetes mellitus; PAD, peripheral artery diseases; CKD, chronic kidney disease; AF, atrial fibrillation; PCI, percutaneous coronary intervention; CABG, coronary artery bypass grafting; VA, vasoactive agents; APT, antiplatelet therapy; BB, beta blockers; RASi, renin-angiotensin-aldosterone system inhibitors; Hb, hemoglobin; Plt, platelet; Scr, serum creatinine; TB, total bilirubin; Cl, chlorine; ALT, alanine aminotransferase. Alb, albumin; Lym, lymphocyte count; HDL, high-density lipoprotein cholesterol; TC, total cholesterol; NewTC, non-high-density lipoprotein cholesterol (calculated as TC - HDL-C); mCONUT, modified controlling nutritional status scoring. | | | | |  |
