# Supplementary material for: Prognostic stratification in myocardial infarction using the modified CONUT score: a multidimensional biomarker from the MIMIC-IV cohort
Source: Front Cardiovasc Med. 2025 Jul 24;12:1596575. doi: 10.3389/fcvm.2025.1596575 (PMC12328424; doi:10.3389/fcvm.2025.1596575)
Supplement: Supplementary file 2 [file Datasheet1.pdf]

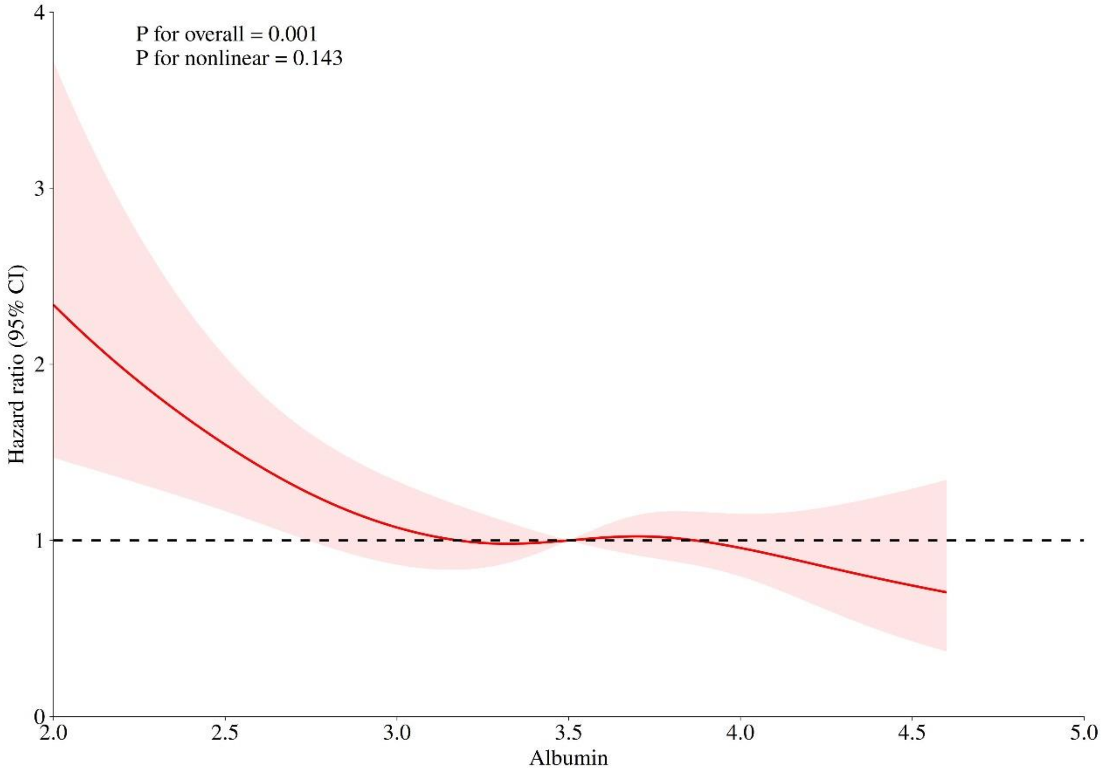

Supplementary Figure 1. Restricted Cubic Spline Analysis of Albumin Levels and Hazard Ratio (Overall P=0.001; Nonlinear P=0.143). A Significant Linear Association Is Observed Without Evidence of Nonlinear Effects.

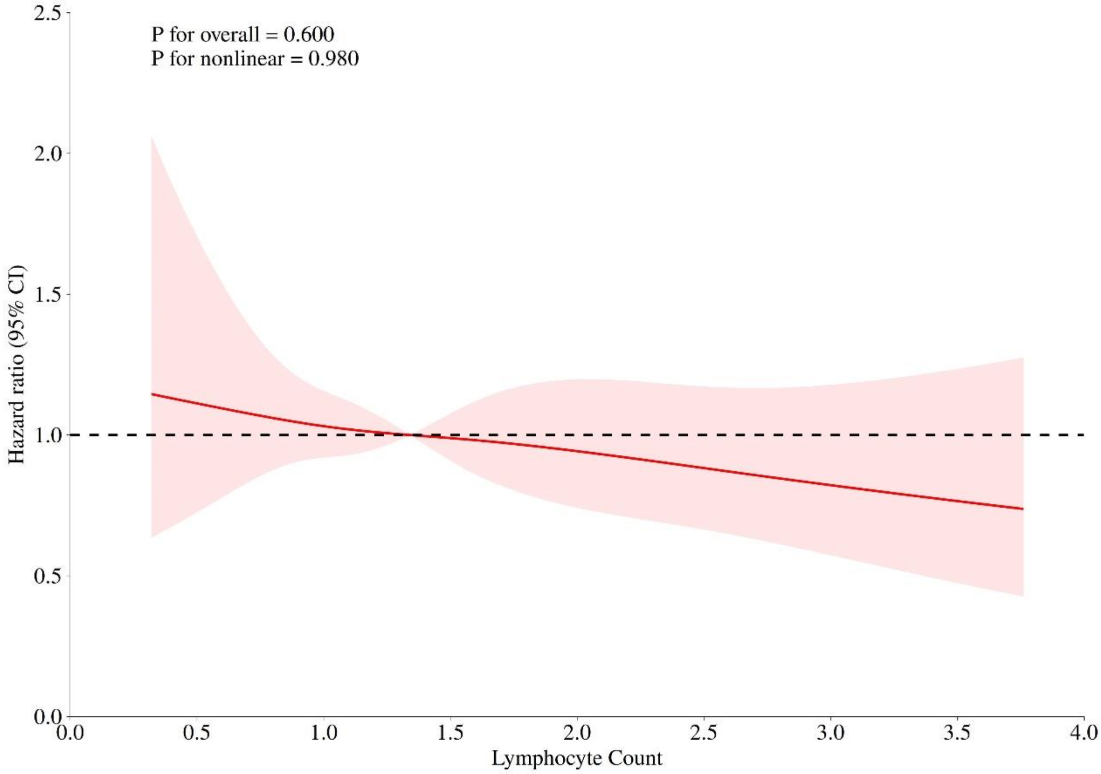

Supplementary Figure 2. Lymphocyte Count and Hazard Ratio (Overall P=0.600; Nonlinear P=0.980). No Significant Associations Observed Across the Measured

Range.

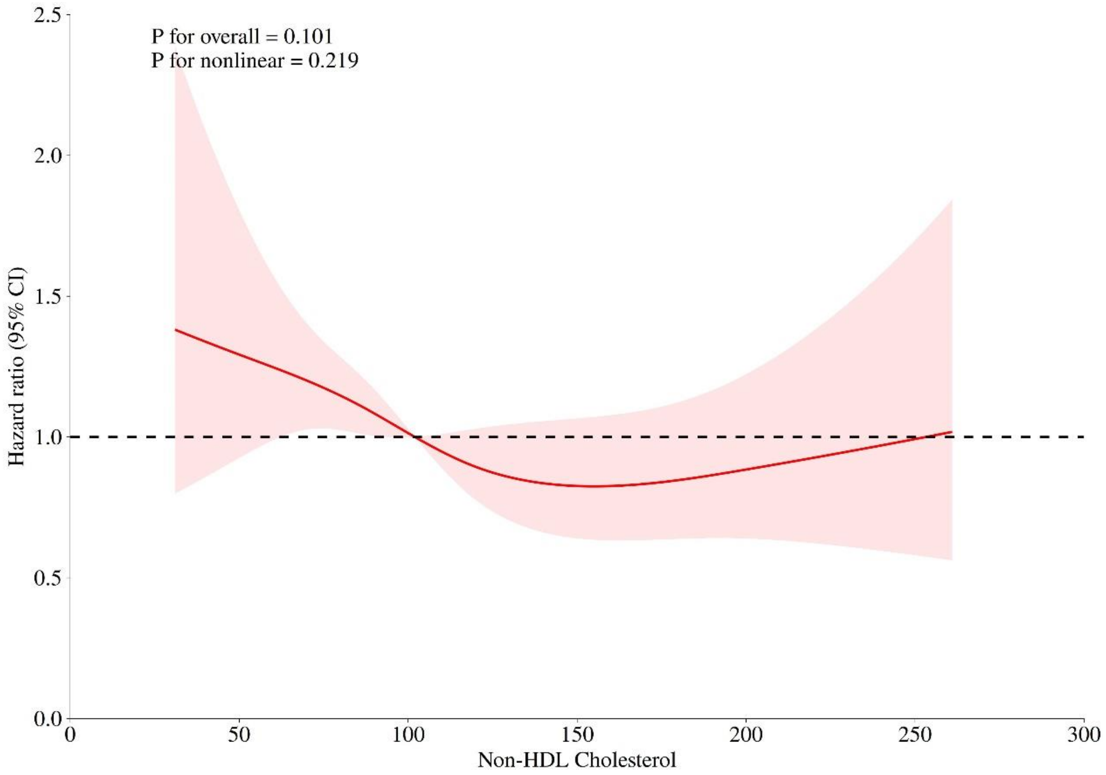

Supplementary Figure 3. Non-HDL Cholesterol and Hazard Ratio (Overall P=0.101; Nonlinear P=0.219). No Statistically Significant Linear or Nonlinear Trends Identified.
